# The role of exercise in improving hyperlipidemia-renal injuries induced by a high-fat diet: a literature review

**DOI:** 10.7717/peerj.15435

**Published:** 2023-06-01

**Authors:** Jun Shunzi Chen, Peng Fei Xie, Hong Feng

**Affiliations:** 1 Institute of Exercise and Health, Tianjin University of Sport, Tianjin, Tianjin, China; 2 Institute of Physical Education, Guiyang University, Guiyang, Guizhou, China; 3 Guizhou Institute of Sports Science, Guiyang, Guizhou, China

**Keywords:** High fat diet, Hyperlipidemia-renal injury, Exercise, Lipotoxicity, Oxidative stress, Endoplasmic reticulum stress, Inflammatory reaction, Nursing

## Abstract

A diet that is high in sugar and fat is a precursor to various chronic diseases, especially hyperlipidemia. Patients with hyperlipidemia have increased levels of plasma free fatty acids and an ectopic accumulation of lipids. The kidney is one of the main organs affected by this disease and, recently, there have been more studies conducted on renal injury caused by hyperlipidemia. The main pathological mechanism is closely related to renal lipotoxicity. However, in different kidney cells, the reaction mechanism varies due to the different affinities of the lipid receptors. At present, it is believed that in addition to lipotoxicity, hyperlipidemia induced-renal injury is also closely related to oxidative stress, endoplasmic reticulum stress, and inflammatory reactions, which are the result of multiple factors. Exercise plays an important role in the prevention of various chronic diseases and recently emerging researches indicated its positive effects to renal injury caused by hyperlipidemia. However, there are few studies summarizing the effects of exercise on this disease and the specific mechanisms need to be further explored. This article summarizes the mechanisms of hyperlipidemia induced-renal injury at the cellular level and discusses the ways in which exercise may regulate it. The results provide theoretical support and novel approaches for identifying the intervention target to treat hyperlipidemia induced-renal injury.

## Introduction

Improved living standards have caused dramatic changes in the diet and lifestyle of modern populations (An et al., 2020). As a result, hyperlipidemia is becoming more and more common due to the excessive intake of sugar and fat, combined with a sedentary lifestyle (Berberich & Hegele, 2022). Hyperlipidemia is a chronic disease marked by abnormal cholesterol and/or triglycerides (Nelson, 2013). As the disease progresses, hyperlipidemia can become secondary to atherosclerosis, obesity, coronary heart disease, fatty liver, stroke, diabetes, hypertension, sudden cardiac death, and other diseases (Afshin et al., 2017; Barrios et al., 2017). In 2017, approximately 3.9 million people died with high levels of non-high-density lipoprotein cholesterol, half of which lived in East Asia, Southeast Asia, and South Asia (Collaboration, 2020). Approximately 93 million people (≥ 20 years old) in the United States had abnormal cholesterol in 2021 (Virani et al., 2021). The prevalence of dyslipidemia among young adults (18–45 years old) is increasing by the year, and abnormal levels of high-density lipoproteins in the southeast coast of China was 22.9% in 2020 (Zhang et al., 2020).

Hyperlipidemia damages the internal organs, especially the kidneys. Many studies have shown that patients with chronic kidney disease often have dyslipidemia, as well (Attman, Samuelsson & Alaupovic, 1993; Majumdar & Wheeler, 2000). Nearly half of these patients have abnormally high triglycerides (TG), and about a third have abnormally high levels of total cholesterol (TC) and low-density lipoprotein cholesterol (LDL-C) (Burst & Benzing, 2011). In addition, research has shown that TC and LDL-C levels are positively correlated with proteinuria levels, and negatively correlated with plasma osmotic pressure in patients with chronic kidney disease (Joven et al., 1996). Hyperalbuminuria and low plasma osmotic pressure are related to apolipoprotein *β* (the main protein in LDL-C). The mechanism of hyperlipidemia-renal injury has become a recent topic of interest to many researchers. Since Moorhead et al. (1982) put forward the “lipid nephrotoxicity hypothesis” in 1982, increasing evidence has supported the idea that lipid abnormalities can lead to glomerulosclerosis and renal interstitial related diseases (Liu, 2011; Tomiyama-Hanayama et al., 2009). However, the mechanism by which hyperlipidemia causes proteinuria (Gutwein et al., 2009; Mallela et al., 2019; Zhao et al., 2019), glomerulosclerosis (Hara et al., 2015), or interstitial disease (Khan et al., 2018; Li et al., 2018) in the kidney is still contested. At present, it is believed that it may be closely related to endoplasmic reticulum stress, inflammatory stress, or oxidative stress (Ruan, 2018).

Exercise has been recommended as an auxiliary treatment for many modern chronic diseases, and may improve the lifestyle of chronic kidney disease patients. Exercise may regulate blood lipid metabolism and significantly decrease TG, TC, and LDL-C values (Banz et al., 2003; Hui et al., 2015). Recently, Qian et al. (2021) found that exercise also protects against renal damage caused by hyperlipidemia. The effects of exercise on hyperlipidemia-renal injury have gradually emerged, but the specific mechanism remains to be explored (Qian et al., 2021; Wang et al., 2021).

Few studies have examined the mechanisms of hyperlipidemia-renal injury in terms of different kidney cells, which have different functions, and the injury mechanism in the disease (Kang & Zhang, 2022; Ruan, 2018). Additionally, the role of exercise is not clear and the regulatory mechanism has only been partially studied (Qian et al., 2021; Wang et al., 2021). As more patients are afflicted by this disease, it is important to understand the pathogenesis and pathomechanism of hyperlipidemia-renal injury from the perspective of different cells in order to find appropriate therapeutic targets. Additionally, the exercise’s regulatory mechanisms should be fully understood for disease prevention and treatment purposes in the future.

Therefore, this article summarizes our understanding of hyperlipidemia-renal injury and the possible regulation mechanism of exercise. The etiology of lipid disorders from macro lipid deposition to micro renal cells, as well as the impact of the endoplasmic reticulum, inflammation, and oxidative stress mechanisms, are described to provide support and new ideas for researchers, doctors, and caregivers in treating this disease.

## Survey Methodology

We searched PubMed, the Web of Science, Embase, Cochrane, the China National Knowledge Infrastructure, WangFang database, CQVIP and Chinese biomedical literature in English or Chinese by three searching strategies in the following: 1. “exercise” as subject heading or its free terms in combination with “hyperlipidemia-renal injury” or its free terms 2. “exercise” or its free terms in combination with “high fat diet/ hyperlipidemia” or their free terms 3. Keywords and subject headings related to “exercise”, “kidney/renal/mesangial cell/endothelial cell/podocyte/renal tubular epithelial cell” and “lipotoxicity/oxidative stress/endoplasmic reticulum stress/inflammatory” were used (the specific searching strategies can be found in the supplemental searching strategies). In addition, I would refer to these references of primary articles. Earlier literature reviews on the mechanisms of hyperlipidemia-renal injury were not miss either (Gyebi, Soltani & Reisin, 2012; Ruan, Varghese & Moorhead, 2009).

### A high fat diet induces lipid deposition in the kidney

A number of animal experiments have shown that a high fat diet (HFD) can cause renal lipid deposition, and subsequently, glomerulosclerosis, tubular fibrosis, and other damage (Szeto et al., 2016; Weihong, Bin & Jianfeng, 2019; Zhou et al., 2022). The study of a HFD is conducted in two ways in animal models: one way is to add an extra proportion of cholesterol to the normal feed (Ding et al., 2022), and the other is to increase the proportion of the energy supply from fat (Jiang et al., 2020). A diet that provides energy by increasing the percentage of fat is also called a high calorie diet. In the normal diet group, lipids, proteins and carbohydrates account for 10%, 20%, and 70% of the total calories, respectively (Jiang et al., 2020). In a high-fat diet, fat, protein and carbohydrate account for 60%, 15%, and 25% respectively (Li et al., 2022b). In animal models, even a diet containing 1% cholesterol has been shown to induce kidney damage (Ding et al., 2022). A low cholesterol concentration is commonly seen in mouse models. Wang et al. (2018) fed mice a 2.5% cholesterol diet, which resulted in hypercholesterolemia, the proliferation of renal mesangial cells, vacuolar injury of renal tubules, and peripheral inflammatory infiltration. However, it is common to add 3%–4% cholesterol to a diet to demonstrate high-fat kidney injuries in the rat model. Kasiske et al. (1990) added 4% cholesterol to the diet of rats and fed them for 14 weeks; their results showed that the renal cortex contained cholesterol esters. Correlation analysis showed that these results were closely related to the development of glomerulosclerosis (*r* = 0.90, *P* < 0.01) and renal tubulointerstitial damage (*r* = 0.64, *P* < 0.05) (Kasiske et al., 1990). In addition, if diet-induced hyperlipidemia is accompanied by the loss of renal function, which is common in nephrectomy models, it leads to a significant increase in the severity of glomerular damage. Kim et al. (2009) found that the rats with HFD containing 4% cholesterol received a unilateral nephrectomy one month later; the rats in the nephrectomy group had more severe glomerular injuries than those in the simple high cholesterol diet group. In animal models, a high fat diet also causes lipid deposition in kidney tissues in a short period of time. Li et al. (2022b) studied mice with a high fat diet for 21 weeks, and selected four-time nodes at nine, 13, 17 and 21 weeks. Their results showed that, compared with the normal diet group, the plasma TC and TG in the HFD group had significantly increased on the ninth week. The renal histology results of HE staining showed that lipid droplets in the renal tubulointerstitium also appeared following the ninth week.

### Effect of lipid accumulation on different cells and its transport mechanism in kidney

**Figure 1:**
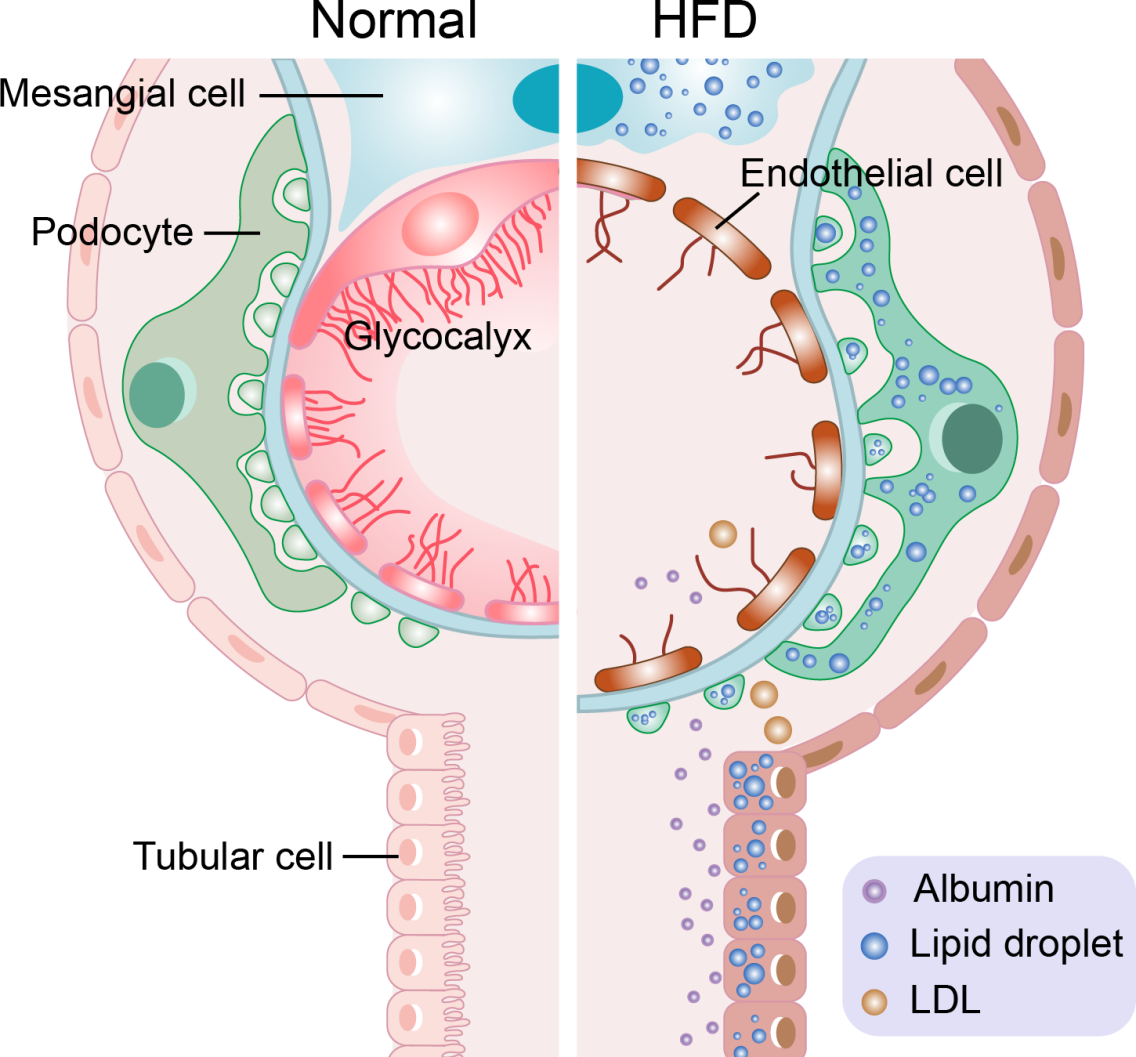
Schematic representation of the lipotoxic reaction of four types of cells in hyperlipidemia-renal injury. Mesangial cells have inflammatory reaction and foam cells formation. The glycocalyx of endothelial cells was destroyed. The slit diaphragm of podocytes was damaged, and podocytes disappearance, shedding, density decreasing and basement membrane abnormality occur. At the same time, albumin cross the damaged glomerular filtration barrier (endothelial cells, basement membrane, podocytes) and proteinuria is produced. Tubular cells loss their brushes and the cross-sectional area of them enlarge.

There are too many free fatty acids (FFAs) in the plasma of patients with hyperlipidemia caused by a high-fat diet, leading to the ectopic accumulation of lipids in the kidney (Bobulescu et al., 2008). When the levels of FFAs are too high in the kidney, its metabolites, such as fatty acyl coenzyme A and ceramide, will accumulate and lead to renal lipotoxicity (Kang & Zhang, 2022; Ruan, 2018). Renal lipotoxicity and the local hypoxia of renal tissue caused by renal extrusion affect renal function (Chade & Hall, 2016). Oxidized low density lipoprotein (ox-LDL) can also cause a renal lipotoxic reaction (Armesilla & Vega, 1994). However, the transport mechanism of excessive FFA and ox-LDL is diverse as is their effect on different kidney cells. The following section introduces the lipotoxic reaction and its mechanism of different cells in terms of four aspects (four kinds of cells). The lipotoxic reaction of different cells can be seen in Fig. 1.

#### Mesangial cells

In 1933, German anatomist Zimmerman (1933) proposed a third kind of cell in the kidney, after epithelial cells and endothelial cells. He named the cell the mesangial cell, which means “container in the middle”. Mesangial cells not only form capillary walls in the glomerulus, but also extend to areas outside the glomerulus to form the “stem” of the glomerulus, which is called extraglomerular mesangium (Lemley & Kriz, 1991). Mesangial cells and the surrounding matrix form the mesangium, which is an important part of the glomerulus. Mesangial cells can regulate the circulation of capillaries and affect glomerular hemodynamics due to their contractile properties similar to smooth muscle cells and a variety of vasoactive substance receptors (Kurihara & Sakai, 2017; Schlöndorff & Banas, 2009). Mesangial cells also perform endocytosis to remove the protein retained in the glomerular filter and promote the renewal of glomerular basement membrane (Baud et al., 1983). With HFD-feeding, mesangial cells will continue to proliferate, causing the secretion of inflammatory factors and the production of the glomerular matrix (including type IV collagen, fibronectin, laminin, and other extracellular matrix components) (Chaudhari et al., 2020; Shotorbani et al., 2020). He et al. (2017) showed that rats fed with a high-fat diet for 22 weeks showed proteinuria, glomerular hypertrophy, and mesangial cell proliferation compared with those fed a normal diet. In addition, the expression of interleukin 6 (IL-6) and tumor necrosis factor α (TNF-α) increased at both the mRNA and protein levels in the high-fat diet group. A large number of low-density lipoprotein receptors (LDLr) was also expressed in glomerular mesangial cells (Chen et al., 2007), which can mediate the endocytosis of low-density lipoproteins (LDL). Several studies have shown that IL-1β can promote the uptake of ox-LDL in mesangial cells, which causes the excessive synthesis of cell cholesterol and decreased outflow, resulting in an imbalance of cholesterol metabolism (Liu et al., 2020; Yang et al., 2017a). Therefore, in a high-fat environment, inflammatory factors also promote the uptake of lipids in mesangial cells, resulting in lipid deposition and the formation of foam cells, which ultimately lead to glomerulosclerosis (Erbay et al., 2009).

Bruneval et al. (2002), however, found mesangial dilation in his HFD ApoE-/- rat model, showing that some glomeruli had obvious diffuse or focal mesangial dilation. Even if mesangial expansion was found, there was no significant change in the proliferation of glomerular cells compared with the normal diet group, indicating that there was no proliferation of glomerular mesangial cells. Moreover, recognized markers of mesangial cell activation in the HFD group, α-smooth muscle actin, did not change significantly either. This effect may be due to the difference between mesangial expansion and mesangial cell activation (Joles et al., 2000). Mesangial expansion is not only a case of mesangial proliferation, but may also be the recruitment and activation of macrophages in the mesangial area (Li et al., 2022a). Bruneval et al. (2002) believed that endothelial cells, not mesangial cells, in the renal mesangial area were closely related to the recruitment of macrophages. Macrophage mediated glycolipid toxicity is achieved through the combination of highly-expressed myeloid related protein-8 (MRP8) and toll-like receptor 4 (TLR4) in target cells. TLR4 is expressed when mesangial cells, endothelial cells, and podocytes are damaged (Brown et al., 2007; Pawar et al., 2009). Kuwabara et al. (2014) found that mesangial cells, endothelial cells, and podocytes are involved in the recruitment of macrophages.

#### Endothelial cells

Located in the inner wall of blood vessels is a separate layer of cells called endothelium, which form a barrier between blood and surrounding tissues. The surface area of the human endothelium is approximately 4,000–7,000 m^2^, and different endothelium can be divided into 1–6 × 1,013 endothelial cells (Jaffe, 1987). Endothelial cells have different appearances and functions as a result of their different positions. The glomerular endothelium in the kidney, like other endothelia, can regulate vascular tension, blood coagulation, and blood cell transport. In addition, the glomerular endothelial cells are arranged in a continuous manner and there are many special windows on the surface, with the aperture of 60–80 nm. The endothelial surface is covered with a negatively charged polysaccharide protein complex gel-like layer, called glycocalyx (Dane et al., 2013). The existence of these special structures is the reason endothelial cells constitute the first filtration barrier of the glomerulus, with high permeability to water molecules and selective filtration of other solute molecules (Deen, Lazzara & Myers, 2001). As cells directly exposed to the blood circulation, glomerular endothelial cells are vulnerable to pathogenic factors in the blood. This exposure causes the destruction of the window structure and sugar calyx, reduces the cell’s defenses, and results in proteinuria. Eventually this leads to primary or secondary glomerular diseases (Jeansson & Haraldsson, 2006). Hypercholesterolemia can induce or enhance the activation of endothelial cells, indicating that endothelial cells express cell surface adhesion molecules, including VCAM-1 and ICAM-1 (Kułdo et al., 2013). The activation of endothelial cells induces the proliferation of smooth muscle cells, platelet aggregation, oxidation of low-density lipoprotein, and atherosclerosis-like changes in the blood vessels (Liao, 2013). Yin et al. (2016) found that lipids accumulated in the kidney in HFD-mice and believed that it was related to the disorder in lipid metabolism of glomerular endothelial cells. The accumulation of lipids may include an increase in the synthesis of cholesterol and fatty acids and/or a decrease in the outflow of cholesterol. ATP-binding cassette transporter A1 (ABCA1), known as the gatekeeper of cholesterol reversal, promotes the outflow of intracellular cholesterol and phospholipids, and plays an important role in the initiation of HDL production (Attie, 2007; Wang et al., 2001). Previous studies have shown that ABCA1 expression contributes significantly to maintaining cholesterol homeostasis in endothelial cells. It has atherosclerotic protection in the cholesterol efflux pathway (Vaisman et al., 2012; Westerterp et al., 2016). Yin et al. (2016) believed that the decreased expression of ABCA1 fed with a HFD may be the key factor to link lipid accumulation with renal injury in the glomerular endothelial cells of mice. Bruneval et al. (2002) believed that a HFD could cause severe hyperlipidemia and activate glomerular endothelial cells. The activation of endothelial cells may be involved in the recruitment of monocytes into the mesangial region and their transformation into macrophages (Sassy-Prigent et al., 2000). The constant stimulation of lipid accumulation and foam cell formation may lead to inflammation and the development of glomerulosclerosis (Bruneval et al., 2002).

#### Podocytes

Podocytes are regarded as the last gatekeeper of the glomerular filtration barrier. They are highly differentiated epithelial cells located outside the glomerular basement membrane. Podocytes are composed of a cell body and many processes that protrude from the cell body (also known as foot processes (FPs)) (Mundel & Shankland, 2002). The FPs contain a complicated actin cytoskeleton structure. One part of the FP supports podocytes attached to the outside of the basement membrane (Kreidberg et al., 1996), and the other part forms a slit diaphragm (SD) complex, which is considered to be the main limiting site of plasma protein filtration in the podocytes (Yu et al., 2018). The SD is the smallest and most vulnerable part of the three glomerular filtration barriers, so it is necessary to maintain the integrity of podocyte structure and function to prevent glomerular albumin leakage. Podocytes also secrete type IV collagen and fibronectin, the main components of basement membrane, as well as matrix metalloproteinases (MMPs) related to extracellular matrix remodeling (Naylor, Morais & Lennon, 2021). Damage to the podocytes may have the following results: podocyte disappearance, podocyte shedding, a decrease in podocyte density, and basement membrane abnormality (Barutta, Bellini & Gruden, 2022).

**Figure 2:**
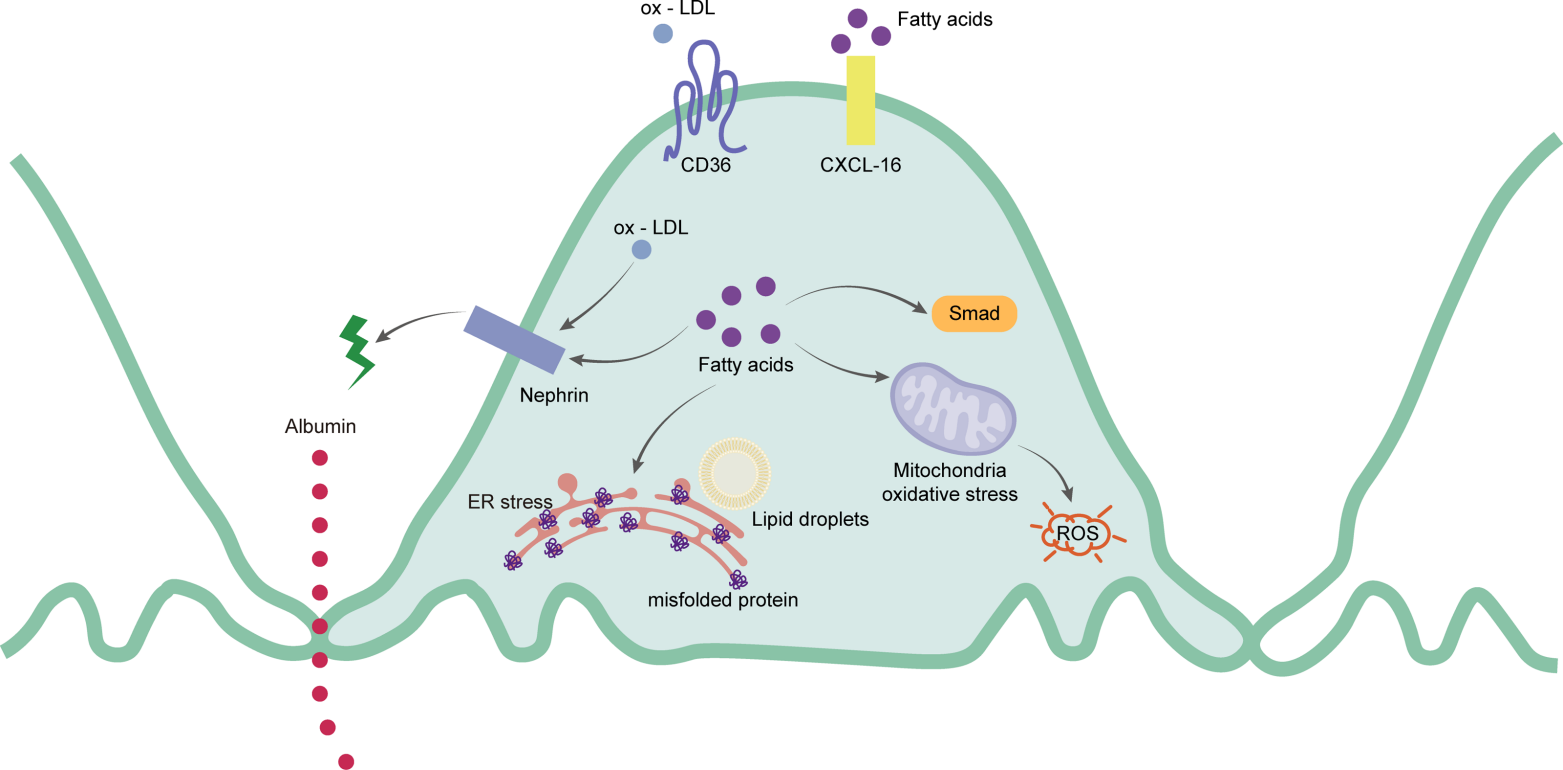
Schematic representation of the mainly mechanism of podocytes in hyperlipidemia-renal injury. Ox-LDL and fatty acids can be captured by CD36 and CXCL-16 separately. Ox-LDL will cause the expression of nephrin decreased, which leads to the destruction of slit diaphragm and albumin leakage. Excessive fatty acids will not only destroy slit diaphragm but also cause activation of Smad pathway, ROS and mis-folded proteins production.

Podocytes are extremely sensitive to lipid metabolism disorders. Excessive lipid accumulation in podocytes lead them to express a variety of lipid-related genes as well as to improve the homeostasis of intracellular lipid metabolism by interfering with the process of lipid synthesis and decomposition, and uptake and outflow (Wahl, Ducasa & Fornoni, 2016). Excessive FFA and ox-LDL will affect the lipid metabolism of podocytes. CD36 is a multifunctional transmembrane glycoprotein that can mediate the uptake of FFA and ox-LDL (Armesilla & Vega, 1994). It is highly expressed in podocytes, renal tubular epithelial cells, mesangial cells, endothelial cells, and interstitial macrophages (Hua et al., 2015; Kennedy et al., 2013; Yang et al., 2017b). CD36 increases the absorption rate of FFA by promoting intracellular metabolism in podocytes (Xu et al., 2013). When FFA accumulates in podocytes, the accumulated FFA is trapped in the mitochondrial matrix, causing increased ROS production and lipid peroxidation, ultimately resulting in mitochondrial damage and dysfunction (Schrauwen & Hesselink, 2004). At the same time, the functional marker of the podocytes, called nephrin, decreases. The structure of SD is damaged and the destruction of the barrier increases the permeability of the podocytes. The albumin filtration increases, eventually leading to albuminuria (Cui et al., 2015). Sun et al. (2015) also showed that under a HFD, the abnormal FFA metabolism in podocytes led to the up-regulation of Smad related pathways, contributing to renal fibrosis and glomerulosclerosis. In human podocytes, the chemokine CXCL-16 is the main scavenger receptor of ox-LDL (Gutwein et al., 2009). The accumulation of ox-LDL is captured by CXCL-16, absorbed in the podocytes, and downregulates the protein expression of nephrin, thus affecting the rearrangement of the podocyte skeleton structure and increasing the excretion of albumin (Bussolati et al., 2005). In addition to endothelial cells, ABCA1 also affects cholesterol efflux in podocytes. Studies have shown that the occurrence of ABCA1 deficiency in podocytes also leads to an increase in the content of cardiolipin (CL), further resulting in mitochondrial dysfunction (Brooks-Wilson et al., 1999). Therefore, the down-regulation of ABCA1 expression can be seen in human podocytes with lipid deposition (Herman-Edelstein et al., 2014). This results in the accumulation of cholesterol and cardiolipin in mitochondria of podocytes, leading to mitochondrial dysfunction, and ultimately, to cell apoptosis (Ducasa, Mitrofanova & Fornoni, 2019). Figure 2 shows the main mechanism of the podocytes.

#### Renal tubular epithelial cells

Renal tubular epithelial cells (rRTECs) are located in the outer layer of the renal tubules, and can selectively reabsorb various substances in the tubule fluid through active, passive, or swallowing functions. In addition, epithelial cells are able to secrete and excrete, playing a key role in regulating the balance of water, electrolytes, and acid–base, and maintaining the homeostasis of the internal environment (Kato et al., 2022). The epithelial cells can obtain lipids from blood circulation and glomerular filtrate simultaneously, is sensitive to changes in lipid metabolism, and is prone to damage (De Vries et al., 2014). The excessive accumulation of lipids may cause the flattening of rRTECs, the loss of the brush border, and tubulointerstitial fibrosis (Sun et al., 2020). Similar to podocytes, CD36 plays an important role in the lipid metabolism of rRTECs (Okamura et al., 2007). CD36 affects lipid metabolism by acting as an ox-LDL receptor. When ox-LDL enters into rRTECs, it induces the up-regulation of heme oxygenase (an enzyme that is sensitive to oxygen consumption and reduction in proteinuria nephropathy), causing abnormal intracellular iron metabolism and cell dysfunction.

**Figure 3:**
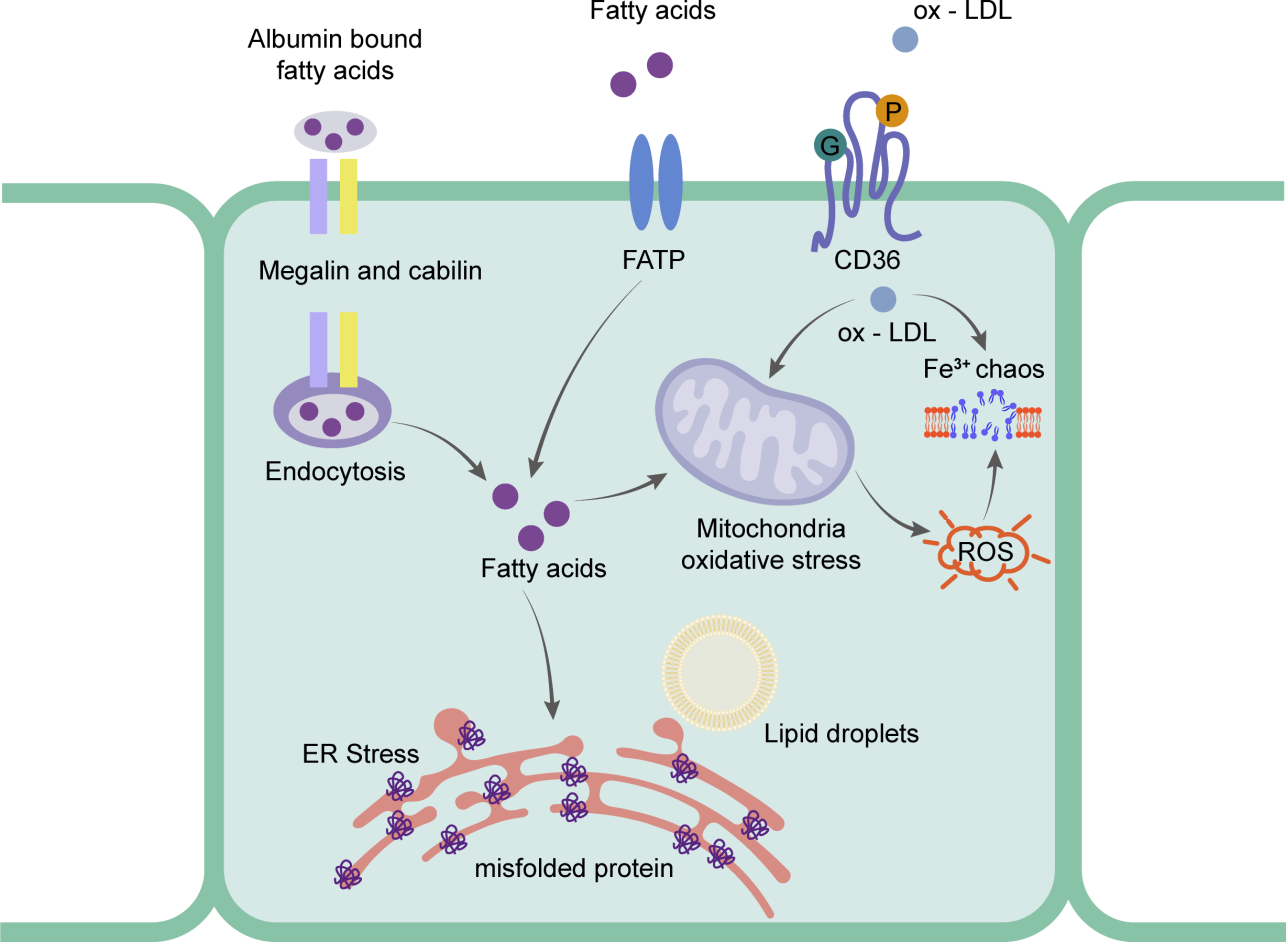
Schematic representation of the mainly mechanism of rRTECs in hyperlipidemia-renal injury. Ox-LDL can be captured by CD36 and absorbed in the cells, which will cause iron chaos and ROS production. Fatty acids will transport by FATP and endocytosis. The excess fatty acids in the rRTECs will finally lead to ER stress and oxidative stress.

Research has focused on the uptake of FFA in rRTECs. Bobulescu et al. (2008) found that plasma FFA was significantly higher than that of a normal diet group by feeding two different species of mice with a high-fat diet for 12 weeks. The accumulated fat was detected in the renal cortex. The cell experiment showed that the higher the concentration of FFA, the higher the percentage of apoptosis. To further prove the direct effect of fat, they found that the intracellular lipid deposition was reduced and the cell function was partially restored by removing FFA from the culture medium (Bobulescu et al., 2009). rRTECs, a kind of cell with a high energy demand, rely heavily on FFA oxidation for energy supply (Kang et al., 2015). Research shows that the number of mitochondria in rRTECs is higher than that in other types of kidney cells (Bhargava & Schnellmann, 2017), which undoubtedly provides a good structural basis for the biological oxidation of FFA in mitochondria. The vast majority of plasma FFA is carried by albumin and the unbound FFA is less than 0.01% (Frayn, Summers & Fielding, 1997). The proximal tubule recycles albumin bound FFA from the filtrate through macroglobulin- and cubilin-mediated albumin endocytosis (Birn & Christensen, 2006; Pollock & Poronnik, 2007). Under normal physiological conditions, FFA carried by recycled albumin contributes to the overall energy balance of the proximal tubules. When the intake of FFA exceeds the demand for energy metabolism, FFA is esterified with glycerol and deposited in lipid droplets in the form of triglycerides. Recent studies have shown that CD36 -/- mice had abnormal blood lipid and proteinuria, and the expression of macroglobulin did not change (Baines et al., 2012). Li et al. (2018) also found that the expression levels of CD36 and NLRP3 in the proximal tubule cells of mice fed with HFD were significantly higher than those in the normal diet group. In experiments, the activation of NLRP3 inflammatory bodies and cell death were induced by palmitate (a saturated fatty acid) by blocking CD36, which suggested that the FFA-induced renal tubulointerstitial damage might be caused by the CD36-NLRP3 inflammatory body pathway. In addition, SLC27 A2 (FATP) also mediates FFA uptake. FATPs (SLC27) is a transmembrane protein family that includes six family members. FATP2 was only expressed in the kidney and liver. Its high expression was also detected in the proximal tubules (Johnson, Stahl & Zager, 2005). Khan et al. (2018) found that FATP2 was located in the apical plasma membrane and cytoplasm of the proximal tubules. When the proximal tubule cells of Slc27a2 -/- were exposed to albumin-bound palmitate, most cells were protected from palmitic acid-induced apoptosis. When compared with the wild type, the daily injection of albumin containing palmitate in Slc27a2 -/- mice showed more renal tubular epithelial cells and less interstitial fibrosis. Figure 3 shows the mechanism of rRTECs.

### Other mechanisms of renal injury in hyperlipidemia

Since Moorhead et al. (1982) put forward the hypothesis of lipid nephrotoxicity in 1982, more experimental research has supported this hypothesis. Lipid abnormalities have been shown to lead to renal atherosclerosis-like changes such as those in glomerulosclerosis and tubulointerstitial diseases. A series of reactions secondary to dyslipidemia trigger multiple mechanisms in the kidney to amplify the damage effect, which includes the activation of oxidative stress, endoplasmic reticulum stress, and inflammatory stress.

#### Oxidative stress

Oxidative stress is a state of imbalance between oxidation and antioxidation; the oxidation reaction is predominant (Qiao, Chen & Zhang, 2023). The mechanism by which hypercholesterolemia and hyperlipidemia promote systemic oxidative stress is unclear. Oxidative stress appears in the early stages of renal injury, which is significantly important for the occurrence and development of high-fat induced renal injury (Scheuer et al., 2000). Research shows that in the rabbit model of hypercholesterolemia induced by a high cholesterol diet, the number of polymorphonuclear leucocytes in the plasma, representing the activity of oxygen free radicals and the malondialdehyde (MDA), a lipid peroxide product, are significantly higher than those in the normal diet group (Prasad & Kalra, 1993). These results suggest that more oxygen free radicals are produced in hypercholesterolemia, resulting in lipid peroxidation damage (Prasad & Kalra, 1993). Scheuer et al. (2000) also showed that xanthine oxidoreductase (XO) activity in the plasma of rats with kidney damage induced by HFD increased compared with the normal diet group, which indicated that the production of oxygen free radicals increased.

The main source of ROS in cells is the NADPH oxidase protein family. This family contains seven members. Four of the members (NOX1, NOX2, NADPH oxidase 4, and NADPH oxidase 5) are considered to be related to renal oxidative stress (Sedeek et al., 2009). NOX4 may be closely related to ROS production with a HFD (Ruggiero et al., 2011). The production of excessive ROS may cause kidney damage in terms of three aspects: (1) it directly acts on DNA, proteins, and lipids to cause damage; (2) it changes the original LDL into ox-LDL, which is absorbed by various types of cells in the kidney. Ox-LDL not only induces apoptosis in the kidney through cytotoxicity, but also stimulates the secretion of a variety of inflammatory factors, leading to the recruitment and activation of macrophages. In addition, ox-LDL, as an activator of NADPH oxidase, promotes ROS production and accelerates LDL oxidation to ox-LDL; (3) it attacks the mitochondria and causes dysfunction.

Some ROS may attack mitochondrial cardiolipin, resulting in the loss of the mitochondrial cristae structure (Szeto et al., 2016). In addition, *in vitro* and *in vivo* evidence shows that damaged mitochondria leak a large amount of cytochrome c into the cytoplasm. The reduction of cytochrome c can disrupt the electron transfer from complex III to complex IV, resulting in increased reverse electron flow and superoxide anion release (Bin et al., 2017; Kartha et al., 2008).

Mitochondria are vulnerable to ROS attack, as are the sites where ROS is generated. HFD can cause excessive production of mitochondrial ROS, leading to an increase in the number of mitochondria splitting into small, fine parts, a decrease in mitochondrial membrane potential, and the induction of apoptosis (Sun et al., 2020).

Current research shows that the kidney, as a high-energy metabolic organ, is rich in mitochondria, which normally rely on fatty acid oxidation for their energy supply. With excessive lipids flowing into the kidney to increase the metabolic load, metabolic reorganization will occur. In other words, the fatty acid oxidative metabolism is reduced or impaired, and glycolysis metabolism increases (Cai et al., 2020). This may increase the aggregation of mitochondrial lipid droplets, promote the destruction of the mitochondrial cristae structure, and cause mitochondrial swelling (Afshinnia et al., 2019).

#### Endoplasmic reticulum stress

The endoplasmic reticulum (ER) is involved in protein folding and post-translational modification. Under normal circumstances, the protein is correctly folded and leaves the endoplasmic reticulum to travel to the Golgi apparatus and other destinations in the cell. Under some internal and external factors, proteins are misfolded or not folded in the endoplasmic reticulum and these proteins accumulate in the endoplasmic reticulum to produce endoplasmic reticulum stress (Rasheva & Domingos, 2009). The unfolded protein response (UPR) is a signal and a protective mechanism of ER stress, which can help the ER process the accumulated misfolded or unfolded proteins to maintain the ER function. In renal diseases, UPR can maintain the homeostasis of the ER by dynamically increasing the ER size and volume (Cybulsky, 2017). The role of UPR mainly depends on the synergism of three effector proteins-mediated signal pathways, namely inositol-requiring enzyme 1 α (IRE1 α), activating transcription factor 6 (ATF6), and protein kinase RNA like ER kinase 2 α kinase (PERK). Under normal physiological conditions, these effector proteins bind to the binding immune protein (BiP) on the ER in an inactive form. Once ER stress occurs, effector proteins separate from the BiP, prompting these effectors to send signals to mediate the relevant signal pathways and rebuild ER homeostasis (Cnop, Foufelle & Velloso, 2012). Hyperlipidemia-related diseases are closely related to the ER stress response. HK-2 cells represent an ER stress reaction which is manifested as the up-regulation of the BiP, IRE1 α and PERK-mediated pathway-related proteins when incubated with the saturated free fatty acid palmitic acid or cholesterol (Qiu et al., 2018). The ER stress caused by similar lipotoxicity was also verified in the ER of podocytes (Park, Han & Kim, 2017; Sieber et al., 2010). When severe ER stress occurs and the unfolded protein defense reaction cannot balance, it will trigger the apoptosis signal pathway. The C/EBP homologous protein (CHOP) is a specific branch of UPR, which is regulated by a variety of transcription factors and triggers apoptosis (Zinszner et al., 1998). Compared with the normal group, HK-2 cells exposed to high concentrations of palmitic acid have increased CHOP expression and ROS production, decreased cell activity, and an increased percentage of apoptotic cells (Li et al., 2019). However, research has shown that pro-apoptosis signals were activated in the renal cortex of HFD mice, and TUNEL positive cells in the kidney were increased (Li et al., 2019).

#### Inflammatory stress

Inflammatory factors may participate with lipids to promote the development of renal injury. Some inflammatory markers, such as C-reactive protein (CRP), interleukin-6 (IL-6), and tumor necrosis factor- α (TNF-α) can be used as a prognostic indicator for patients with chronic kidney disease (Cachofeiro et al., 2008). Cachofeiro et al. (2008) found that there were higher levels of plasma CRP, IL-6, and TNF-α in patients with chronic kidney disease than those with the same age control group, and there was a linear correlation between ox- LDL and CRP. Carvalho et al. (2012) found that the CRP levels of renal dialysis patients were higher than in those who were not on dialysis (10.5 ± 6.3 mg/L). These variations indicate that there is a close relationship between kidney disease and inflammation. Inflammation may aggravate the severity of hyperlipidemia-induced renal injury by affecting cholesterol homeostasis, especially LDL-c homeostasis. A retrospective study on patients with kidney disease showed that with the development of chronic kidney disease, plasma LDL-c gradually declines to a nearly normal level (Moorhead et al., 1993). Similar research results were also found in dialysis patients (Liu et al., 2004). The phenomenon of decreasing plasma LDL-c with increasing disease severity is commonly attributed by most experts and scholars as inflammation leading to cholesterol redistribution. Cells maintain the dynamic balance of cholesterol through its synthesis and excretion under normal physiological conditions. HMGCoA reductase (HMGCoA-R) is a rate-limiting enzyme for cholesterol synthesis (Sever et al., 2003). Stimulated by inflammatory factors such as IL-6, IL-1β, and TNF-α, HMGCoA-R activity increases, promotes the mediated intracellular synthesis of cholesterol, and induces intracellular lipid accumulation and foam cell formation (Chen et al., 2007; Chen et al., 2014b). Many studies have confirmed that the mRNA and protein expression of HMGCoA-R in the kidneys of mice increase in an inflammatory environment (Chen et al., 2014a; Ma et al., 2008; Xu et al., 2011). The ABCA1 protein plays an important role in cholesterol efflux. Inflammation can promote the outflow of cholesterol by upregulating the expression of the ABCA1 protein in the kidney (Liu et al., 2020). Inflammatory stress promotes the increase of cholesterol synthesis and the decrease of cholesterol efflux, leading to the accumulation of cholesterol from the blood in the kidney tissue, and finally a decreased level of plasma LDL-c. Therefore, under the condition of high fat-induced renal injury accompanied by inflammation, cholesterol is transferred from the circulating plasma to renal tissue by regulating the distribution of cholesterol.

### Regulation mechanism of exercise on hyperlipidemia-renal injury

Exercise training is currently promoted as being safe and feasible for developing a healthier lifestyle. The 2020 WHO Guide on Physical Activity and Sitting Behavior recommends that all adults (including people with chronic diseases and disabilities) increase physical activity and reduce sedentary behavior. The recommendation calls for engaging in no less than 150 to 300 min of moderate-intensity physical activity per week, or no less than 75 to 150 min of high-intensity physical activity, or a combination of moderate-intensity and high-intensity aerobic physical activity of the same intensity (Jing et al., 2021).

**Figure 4:**
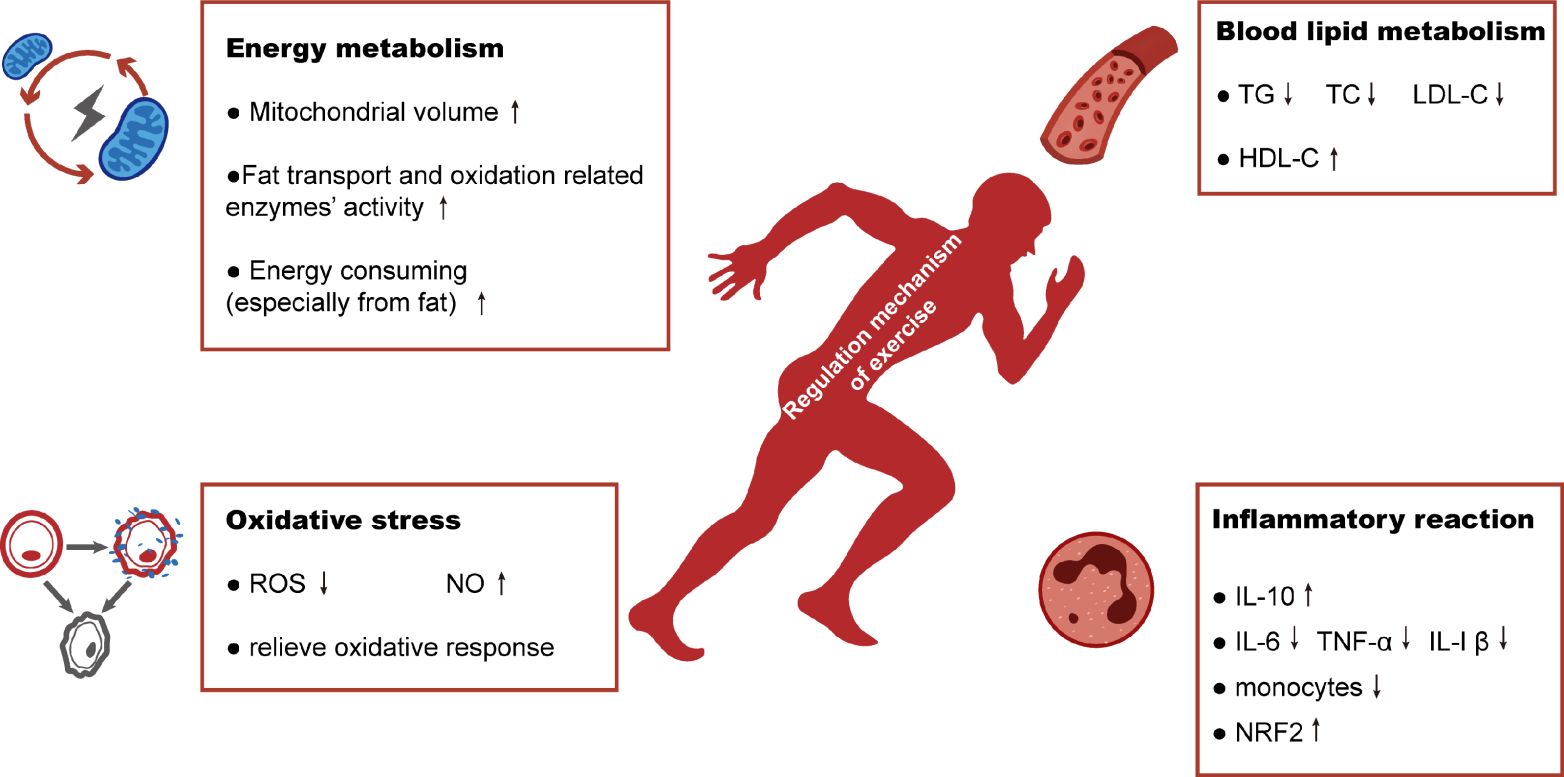
Schematic representation of regulation mechanism of exercise in hyperlipidemia-renal injury. Exercise will improve hyperlipidemia-renal injury possibly mainly by four aspects: increasing energy metabolism, alleviating the abnormalities of blood lipid metabolism and reducing the production of oxidative stress and inflammatory reaction.

Exercise therapy is often used as a safe and effective method to improve the lifestyle of people with hyperlipidemia. In this article we review the four possible mechanisms by which exercise may address high-fat induced-renal injuries (Fig. 4).

#### Regulation on energy metabolism

Energy intake typically exceeds the energy expenditure in a high energy diet, resulting in an energy imbalance. Excess energy is stored in the body as fat. Exercise has been shown to increase energy expenditure by increasing exercise duration or intensity, leading to a reduction in excess adiposity. Exercise can change body composition especially in terms of reducing fat mass. Donnelly et al. (2013) conducted a study of aerobic exercise on overweight patients for 10 months. The subjects in the exercise group expended 400 kcal to 600 kcal every week without limiting energy intake. The results showed that overweight patients in the exercise group lost weight, and their body fat percentage and fat mass significantly decreased. In addition to aerobic exercise, the meta-analysis of Wewege et al. (2017) also showed that high-intensity intermittent exercise promoted the reduction of body fat mass and waist circumference. For people with hypercholesterolemia, exercise can have a similar effect. Santosa et al. (2007) conducted weight loss training over 24 weeks for patients with high cholesterol and their visceral fat and subcutaneous fat decreased significantly. Hawley & Yeo (2013) hypothesized that regular exercise could lead to skeletal muscle adaptation and promote fat oxidation in the entire body. This increased adaptability of the skeletal muscle is the ability of muscle tissue to oxidize fat. It is specifically manifested in the increased oxidation of fat fuel (TG in fat and muscle, and FFAs in the blood), thus reducing glycogen decomposition. The increased adaptability of skeletal muscle may be due to the effect of regular exercise on mitochondria: exercise increases the volume of the mitochondria and the activity of enzymes related to fat transport and oxidation (Holloszy & Coyle, 1984). As a fatty acid transposase, CD36 is also expressed on the mitochondrial membrane and is closely related to the utilization of fatty acids under endurance exercise (Smith et al., 2011). McFarlan et al. (2012) found that the respiratory exchange rate (RER) of skeletal muscle of CD36 -/-mice was higher than that of wild type mice, and the fatty acid transport rate was lower than that of wild type mice. Holloway et al. (2009) also found similar findings that the mitochondrial fatty acid oxidation rate was significantly lower in the CD36 deletion group than in the wild type exercise group. Although its mechanism is not completely clear, it has been shown that the increase of the mitochondrial CD36 protein is related to an increase of the mitochondrial fatty acid oxidation rate.

#### Regulation on blood lipid metabolism

Hyperlipidemia is the driving factor of renal injury. However, its abnormal lipid metabolites may be improved through exercise training. Many studies have shown that aerobic exercise can affect lipids metabolism (Banz et al., 2003; Hui et al., 2015; Leon & Sanchez, 2001), as well as various indicators related to lipid metabolism. Aerobic exercise is a kind of exercise to train cardiorespiratory endurance by means of fast walking, jogging, cycling, *etc.*, (Mann, Beedie & Jimenez, 2014). Aerobic exercise can improve the blood lipids values in patients with hyperlipidemia by reducing the levels of serum TG, TC, and LDL-c, and increasing the HDL-c levels. HDL-c may be the most easily improved lipid component as a result of increased physical activity. Banz et al. (2003) used an aerobic training program (3 times/week on ski style fitness equipment, 85% HRmax, 40 minutes/session) for 10 weeks, and found that HDL-c increased by 13% (from 29.8 to 33.7 mg/dL, *p* < 0.05). Leon & Sanchez (2001) conducted a meta-analysis of aerobic exercise with durations of no less than 12 weeks (*n* = 4,700). On average, HDL-c increased by 4.6%. Hui et al. (2015) found that the level of lipoprotein lipase increased after aerobic exercise, thus contributing to the synthesis of HDL-c. Moderate intensity aerobic exercise may be effective in increasing HDL-c to promote the removal of LDL-c. However, in order to directly reduce LDL-c and TG, exercise intensity needs to be increased. O’Donovan et al. (2005) directly evaluated the impact of training intensity by controlling the amount of training. A total of 64 previously sedentary men were randomly assigned to the control group, medium intensity exercise group (60% VO2max), or high intensity exercise group (80% VO2max). The two exercise groups completed 400 kcal training three times-a-week for 24 weeks. Significant lipid improvement for TC (from 6.02 to 5.48 mmol/L), LDL-c (from 4.04 to 3.52 mmol/L), and non-HDL-c (from 4.58 to 4.04 mmol/L) only occurred in the high-intensity exercise group.

The regulation mechanism of exercise-related lipoproteins, especially the regulation of cholesterol, is closely related to the receptors in the reverse cholesterol transport (RCT) mechanism (Marques et al., 2018). Butcher et al. (2008) showed that aerobic exercise can change the lipid characteristics of long-term sedentary individuals by increasing their HDL-c concentrations. This effect may be due to the activation of the PPAR-γ/LXRα pathway and the positive regulation result from the increased expression of ABCA1 and ABCG1 proteins. Similar findings were also found in animal experiments. After six weeks of aerobic training at 65% VO2max, the expression of ABCA1 in the liver of Wistar rats increased by about 30%, and the concentration of HDL-c, β- HDL and lecithin cholesterol acyltransferase (LCAT) also increased by about 30% (Ghanbari-Niaki et al., 2007). LCAT is a vital enzyme in the synthetic process of HDL-c. As a result of the increasement, excess cholesterol could be removed more effectively *via* RCT. In summary, various evidence support that physical activity possible affects the level of HDL-c easily. Perhaps the core mechanism of the alternation owing to RCT mechanism. As to other lipid components such as LDL-c and TG, high intensity of exercise may be needed and the evidence is limited.

#### Regulation on oxidative stress

An increase in oxidative stress is one of the key characteristics of renal injury in hyperlipidemia. Under normal physiological conditions, the balance between oxidative stress and antioxidant defense maintains organisms’ stability (Blokhina, Virolainen & Fagerstedt, 2003). In hyperlipidemic-kidney injury, this increase in oxidative stress is mainly manifested in the increase of free radicals, which is related to the accumulation of cholesterol in serum and tissues (Napoli & Lerman, 2001). In addition, the increase of oxidative stress is also reflected in the upregulation of key regulatory factors of the oxidative stress response (Loboda et al., 2016). Qian et al. (2021) used ApoE-/- mice to study the effect of 12 weeks of swimming exercise on high-fat induced renal injury. The results showed that the expression of NRF2 in renal tissue was significantly higher in the HFD than that of the normal diet group. After training, the expression of NRF2 decreased, suggesting that NRF2, as a key regulator of the oxidative stress response, increased the level and expression of oxidative stress in the HFD group. Exercise can protect against hyperlipidemia-induced kidney damage by reducing oxidative stress, which, in turn, reduces the concentration of plasma superoxide dismutase (SOD) and glutathione peroxidase (GSH-PX). However, one of the key case mechanisms of hyperlipidemia-renal injury is the excessive production of ROS. Many studies have shown that exercise can reduce ROS production. Ji et al. (2018) found that the ROS level in the kidney tissue of SD rats after aerobic training was significantly lower than that of the control non-exercise group. Similar results were found by Morris et al. (2009), who showed that the ROS levels in the kidney tissue of rats in the high aerobic group was significantly lower than those in the low aerobic group. Besides, Cao et al. (2016) found oxidative stress were attenuated in moderate exercise group, which may attribute to enhance the level of nitric oxide and offer a protective adaptation by affecting antioxidant enzyme gene expression in hypertriglyceridemia kidney damage. Therefore, exercise may play a regulatory role by influencing the production of ROS and NO production and attenuating the oxidative stress reaction.

#### Regulation on inflammatory reaction

The above discussion has shown that high fat diet-induced renal injury is accompanied by inflammation, and that inflammation promotes the transfer of cholesterol from plasma to tissue. A large number of studies have shown that exercise can improve the inflammatory reaction and, in turn, the quality of life of patients with chronic kidney disease or those on renal dialysis (Machado et al., 2017). The results of Ishikawa et al. (2012) suggest that low-intensity exercise can slow the development of diabetic nephropathy by reducing the level of urinary albumin, maintaining the number of podocytes, and reducing inflammation. Liu, Kao & Wu (2019) also found that exercise can alleviate inflammation and improve metabolic dysfunction. In the hyperlipidemia-renal injury model of Qian et al. (2021), the renal tissue showed obvious inflammatory infiltration, collagen deposition, and activation of the fibrosis pathway. However, these effects were significantly alleviated in the exercise group. Exercise may alleviate inflammation by altering the concentration of pro-inflammatory (such as TNF-α, IL-6) and anti-inflammatory cytokines (such as IL-10). The study of Wang et al. (2021) indicated that aerobic exercise may inhibit M1 macrophages (classically activated pathogenic macrophages that can secrete TNF-α, IL-1β and IL-6, promoting an inflammatory response). In addition to aerobic exercise, resistance exercise can also inhibit inflammation. Saud et al. (2021) showed that the level of IL-10 in the renal tissue of rats with chronic kidney disease decreased, while the level of TNF-α and TGB-β increased after eight weeks of exercise. When compared with the non-exercise group, all indicators improved and the inflammatory reaction was alleviated in the exercise group. Except for regulating these inflammatory cytokines, exercise-associated changes in anti-inflammatory immune cells had been proved. The proportion of pro-inflammatory intermediate monocytes decreased in the regular exercise group compared to non-exercising group (Dungey et al., 2017). Wang et al. (2021) observed a similar change. They found that in the HFD group, the expression of monocyte chemoattractant protein-1 (MCP-1) increased in the kidney, thus promoting the expression of adhesion molecules and other inflammatory factors. In addition, NRF2 has a different role in inflammation from its role in oxidative stress. In the previous literatures about resistance exercise (Abreu et al., 2017), the expression of NRF2 mRNA increased after three months’ exercise. Bishop et al. (2023) thought it was a promising observation for following reasons: (1) NRF2 pathway are usually impaired in those patients with chronic kidney disease or kidney failure (Kim & Vaziri, 2010). (2) Nuclear-factor Kappa B would be blocked and reducing the synthesis of inflammatory cytokines such as IL-6 through NRF2 pathway (Ahmed et al., 2017). Therefore, a variety of exercise patterns may alleviate the inflammatory response by regulating the inflammatory factors, immune cells and NRF2 pathway.

## Conclusions

The consumption of a high-fat diet is known to cause hyperlipidemia. The excessive accumulation of plasma lipids eventually leads to the ectopic accumulation of lipids and damage to the kidney. The mechanism of injury is different in each type of kidney cell due to the various affinities of their lipid receptors to different lipids. Current research shows that in addition to the mechanism of lipid toxicity, the hyperlipidemia-renal damage is a compounded result of oxidative stress, endoplasmic reticulum stress, and an inflammatory reaction. The research on hyperlipidemia-renal injury has gained momentum since 1982, however, the related research content is not sufficient. Topics including the signal pathway and related gene expression of various cellular lipotoxicity mechanisms need to be further explored. Exercise has played a vital role in treating various chronic diseases as a non-drug intervention. For patients with hyperlipidemia-renal injury, exercise can not only regulate the inducing factor of hyperlipidemia, it may also improve the symptoms by regulating energy metabolism, oxidative stress, and the inflammatory reaction. At present, there are few studies on improving hyperlipidemia-renal injury by exercise, especially for lipid metabolism and oxidative stress-related signal pathways in the kidney. Therefore, we discussed the mechanisms involved in hyperlipidemia-renal injury and the possible regulatory mechanisms of exercise. These results may provide theoretical support and new targets for preventing hyperlipidemia-renal injury in the future.

##  Supplemental Information

10.7717/peerj.15435/supp-1 Supplemental Information 1 Supplemental Search Strategies We took the strategies of PubMed as an example.

## Additional Information and Declarations

### Data Availability

The following information was supplied regarding data availability: This is a literature review.
