# Supplementary material for: The role of exercise in improving hyperlipidemia-renal injuries induced by a high-fat diet: a literature review
Source: PeerJ. 2023 Jun 1;11:e15435. doi: 10.7717/peerj.15435 (PMC10239619; doi:10.7717/peerj.15435)
Supplement: Supplemental Information 1 — We took the strategies of PubMed as an example. [file peerj-11-15435-s001.docx]

Take the PubMed search engine for example:

The 1^st^ PubMed search strategy:

| 1 | (((((((Exercise[MeSH Terms]) OR (Exercise[Title/Abstract])) OR (Physical Activity[Title/Abstract])) OR (Physical Exercise[Title/Abstract])) OR (Acute Exercise[Title/Abstract])) OR (Isometric Exercise[Title/Abstract])) OR (Aerobic Exercise[Title/Abstract])) OR (Exercise Training[Title/Abstract]) |
| --- | --- |
| 2 | (hyperlipidemia- renal injury[MeSH Terms]) OR ((((hyperlipidemia- renal injury[Title/Abstract]) OR (hyperlipidemia- renal injuries[Title/Abstract])) OR (Kidney Injury in Hyperlipidemia[Title/Abstract])) OR (hyperlipidemia induced kidney damage[Title/Abstract])) |
| 3 | 1 AND 2 |

The 2^nd^ PubMed search strategy:

| 1 | (((((((Exercise[MeSH Terms]) OR (Exercise[Title/Abstract])) OR (Physical Activity[Title/Abstract])) OR (Physical Exercise[Title/Abstract])) OR (Acute Exercise[Title/Abstract])) OR (Isometric Exercise[Title/Abstract])) OR (Aerobic Exercise[Title/Abstract])) OR (Exercise Training[Title/Abstract]) |
| --- | --- |
| 2 | ((((((Diet, High-Fat[MeSH Terms]) OR (Hyperlipidemias[MeSH Terms])) OR (Diet, High-Fat[Title/Abstract])) OR (Hyperlipidemias[Title/Abstract])) OR (High-Fat Diet[Title/Abstract])) OR (Diet, High Fat[Title/Abstract])) OR (High Fat Diet[Title/Abstract]) |
| 3 | 1 AND 2 |

The 3^rd^ PubMed search strategy:

| 1 | (((((((Exercise[MeSH Terms]) OR (Exercise[Title/Abstract])) OR (Physical Activity[Title/Abstract])) OR (Physical Exercise[Title/Abstract])) OR (Acute Exercise[Title/Abstract])) OR (Isometric Exercise[Title/Abstract])) OR (Aerobic Exercise[Title/Abstract])) OR (Exercise Training[Title/Abstract]) |
| --- | --- |
| 2 | (((((((((((kidney[MeSH Terms]) OR (mesangial cells[MeSH Terms])) OR (endothelial cells[MeSH Terms])) OR (podocytes[MeSH Terms])) OR (renal tubular epithelial cell Kidneys[MeSH Terms])) OR (kidney[Title/Abstract])) OR (mesangial cells[Title/Abstract])) OR (endothelial cells[Title/Abstract])) OR (podocytes[Title/Abstract])) OR (renal tubular epithelial cell Kidneys[Title/Abstract])) OR (Kidneys[Title/Abstract])) OR (Renal[Title/Abstract]) |
| 3 | (((((((((((((((((((((lipotoxicity[MeSH Terms]) OR (oxidative stress[MeSH Terms])) OR (endoplasmic reticulum stress[MeSH Terms])) OR (inflammatory[MeSH Terms])) OR (lipotoxicity[Title/Abstract])) OR (oxidative stress[Title/Abstract])) OR (endoplasmic reticulum stress[Title/Abstract])) OR (inflammatory[Title/Abstract])) OR (Antioxidative Stress[Title/Abstract])) OR (Anti oxidative Stress[Title/Abstract])) OR (Oxidative Damage[Title/Abstract])) OR (Oxidative Stress Injury[Title/Abstract])) OR (Oxidative Injury[Title/Abstract])) OR (Oxidative Cleavage[Title/Abstract])) OR (Oxidative DNA Damage[Title/Abstract])) OR (DNA Oxidative Damage[Title/Abstract])) OR (Oxidative[Title/Abstract] AND Nitrosative Stress[Title/Abstract])) OR (Oxidative Nitrative Stress[Title/Abstract])) OR (Nitro Oxidative Stress[Title/Abstract])) OR (Reticulum Stress, Endoplasmic[Title/Abstract])) OR (Stress, Endoplasmic Reticulum[Title/Abstract])) OR (Innate Inflammatory Response[Title/Abstract]) |
| 4 | 1 AND 2 AND 3 |
